# Identification of novel transcription factors regulating secondary cell wall formation in Arabidopsis

**DOI:** 10.3389/fpls.2013.00189

**Published:** 2013-06-11

**Authors:** Hua Cassan-Wang, Nadia Goué, Mohammed N. Saidi, Sylvain Legay, Pierre Sivadon, Deborah Goffner, Jacqueline Grima-Pettenati

**Affiliations:** Laboratoire de Recherche en Sciences Végétales, UMR5546, Centre National de la Recherche Scientifique, Université Toulouse IIIUPS, Toulouse, France

**Keywords:** secondary cell wall, xylem, fibers, transcription factor, Arabidopsis, lignin, co-expression, biofuels

## Abstract

The presence of lignin in secondary cell walls (SCW) is a major factor preventing hydrolytic enzymes from gaining access to cellulose, thereby limiting the saccharification potential of plant biomass. To understand how lignification is regulated is a prerequisite for selecting plant biomass better adapted to bioethanol production. Because transcriptional regulation is a major mechanism controlling the expression of genes involved in lignin biosynthesis, our aim was to identify novel transcription factors (TFs) dictating lignin profiles in the model plant *Arabidopsis*. To this end, we have developed a post-genomic approach by combining four independent *in-house* SCW-related transcriptome datasets obtained from (1) the fiber cell wall-deficient *wat1 Arabidopsis* mutant, (2) *Arabidopsis* lines over-expressing either the master regulatory activator *EgMYB2* or (3) the repressor *EgMYB1* and finally (4) *Arabidopsis* orthologs of *Eucalyptus* xylem-expressed genes. This allowed us to identify 502 up- or down-regulated TFs. We preferentially selected those present in more than one dataset and further analyzed their *in silico* expression patterns as an additional selection criteria. This selection process led to 80 candidates. Notably, 16 of them were already proven to regulate SCW formation, thereby validating the overall strategy. Then, we phenotyped 43 corresponding mutant lines focusing on histological observations of xylem and interfascicular fibers. This phenotypic screen revealed six mutant lines exhibiting altered lignification patterns. Two of them [Bel-like HomeoBox6 (*blh6*) and a *zinc finger TF*] presented hypolignified SCW. Three others (*myb52*, *myb-like TF, hb5*) showed hyperlignified SCW whereas the last one (*hb15*) showed ectopic lignification. In addition, our meta-analyses highlighted a reservoir of new potential regulators adding to the gene network regulating SCW but also opening new avenues to ultimately improve SCW composition for biofuel production.

## Introduction

Plant cells are enclosed in cell walls, which provide them with structural support and regulate growth and differentiation. There are two main types of cell walls: primary cell walls and secondary cell walls (SCWs). Primary cell walls are formed in all plant cells and are composed mainly of cellulose, hemicellulose, and pectin. SCWs are much thicker and are deposited in the inner side of primary cell walls only in some highly specialized tissues and cell types such as xylem vessels and fiber cells. Lignified SCW are the most abundant source of renewable biomass on earth, and are widely used for construction, paper, and energy. In the context of the energetic crisis, lignocellulosic biomass has received growing attention as raw material for the production of second-generation biofuels.

SCWs are composed of cellulose, hemicelluloses, and lignin. The impregnation with lignin renders SCWs waterproof and resistant, allowing water conduction through xylem vessels as well as mechanical support. On the other hand, for lignocellulosic biofuel production, lignin is a major negative factor preventing hydrolytic enzymes from gaining access to cellulose and, as a result limits the saccharification potential. The biosynthetic pathways leading to SCWs formation are highly regulated at the transcriptional level. Tremendous progress has been made during the last decade supporting the existence of a complex hierarchical regulatory network of transcription factors (TFs). Most of those belong to two large TF families: R2R3-MYB and NAC (NAM/ATAF/CUC) (Demura and Fukuda, 2007; Zhong and Ye, 2007; Grima-Pettenati et al., 2012; Wang and Dixon, 2012; Zhong et al., 2012). Some members of the NAC TF family are key regulators of SCW formation in fibers and/or in vessels. This particular SCW related subgroup of NACs include the NAC SECONDARY WALL THICKENING PROMOTING FACTOR 1 (NST1), SECONDARY WALL-ASSOCIATED NAC DOMAIN PROTEIN1 (SND1/NST3), NST2, and the VASCULAR-RELATED NAC DOMAIN (VND6 and VDN7) (Kubo et al., 2005; Mitsuda et al., 2005, 2007; Zhong et al., 2006, 2012; Ko et al., 2007). Over expression of any of these *NACs* led to ectopic lignification in cells that normally contain only primary cell walls (for review, see Grima-Pettenati et al., 2012). A double mutation of *SND1* and *NST1* resulted in loss of SCW in fibers, whereas the simultaneous repression of *VND6* and *VND7* led to a defect in vessel SCW thickenings (Kubo et al., 2005; Zhong et al., 2007b). In the regulatory hierarchical network SDN1, NST1/2, and VND6/7 are first-level master switches controlling downstream TF regulators (Zhong and Ye, 2007; Wang and Dixon, 2012; Zhong et al., 2012). The second layer of regulators includes many MYB TFs (MYB20, MYB42, MYB43, MYB46, MYB52, MYB54, MYB58, MYB69, MYB61, MYB63, MYB83, MYB85, and MYB103) (Zhong et al., 2008; Ko et al., 2009; McCarthy et al., 2009; Zhou et al., 2009; Romano et al., 2012) as well as several other TFs like SND2, SND3, KNAT7, AtC3H14 Zinc finger TF (Zhong et al., 2008; Ko et al., 2009). Some of these are also master regulators since they control the biosynthesis of the three main components of SCW i.e., cellulose, xylan, and lignin. The discovery of this multi-leveled hierarchical regulatory network has been a breakthrough in our understanding of the regulation of the lignified SCW, although it is far from being complete. For instance, only a few TFs characterized hitherto are regulating specifically one of the SCW components, although three MYBs (MYB85, MYB58, and MYB63) were reported to be lignin-specific. In addition, our knowledge of the molecular mechanisms determining the heterogeneous SCW deposition in different cell types, as well as those governing the various patterns of SCW deposition is still very poor. More efforts are needed to get a comprehensive picture of the transcriptional regulation of the SCWs both from a fundamental and an applied perspective.

As a step toward this goal, we searched for novel TFs potentially implicated in the control of lignin deposition. To do this, we set up a post-genomic approach combining four original in-house SCW-related transcriptomic data sets that enabled us to identify 80 candidates belonging to major plant TFs families (i.e., NAC, MYB, bHLH, Zinc finger, HomeoBox, and AP2/ERF). Most of them have not yet been functionally characterized. Histochemical analyses of the corresponding mutants revealed six strong candidates regulating the biosynthesis of lignin and/or the whole SCW biosynthetic program: BLH6 (Bel-like HomeoBox6; AT4G34610), HB5 (AT5G65310), HB15 (AT1G52150), MYB-like TF (AT3G11280), MYB52 (AT1G17950), and Zinc finger TF (AT3G46620).

## Results

### A post-genomic approach to identify novel regulatory genes involved in SCW formation

**Figure 1:**
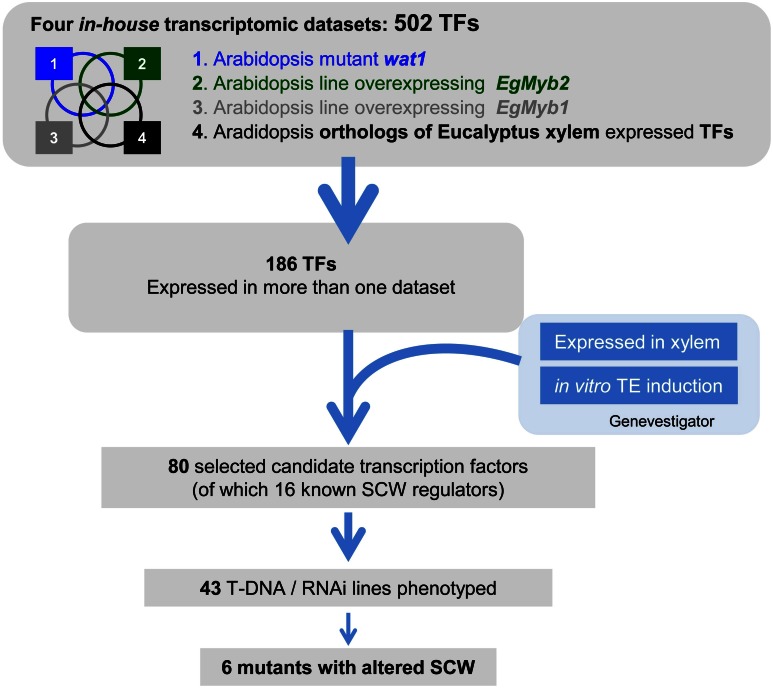
**Overall strategy to identify transcription factors (TFs) involved in SCW formation.** Four in-house SCW formation-related transcriptomic datasets were crossed to select TFs present in more than one dataset/experimental condition. These TFs were further screened against publicly available large-scale transcriptomic datasets, to select those highly or preferentially expressed in the organs and/or tissues of interest. This led to a list of 80 candidate genes, of which we phenotyped the 42 T-DNA insertion mutants and/or RNAi transgenic plants available. The four *in house* SCW related transcriptomic datasets include **(1)** the fiber SCW-deficient *wat1* Arabidopsis mutant, **(2)** Arabidopsis lines over-expressing the SCW master activator EgMYB2, **(3)** Arabidopsis lines over-expressing the SCW master repressor EgMYB1, and **(4)** Arabidopsis orthologs of Eucalyptus xylogenesis-related genes.

In order to identify novel regulatory genes involved in SCW formation, we took advantage of four large scale *in house* transcriptomic data sets and developed a post-genomic approach. A flow chart of the main steps of this original strategy is described in Figure 1. The first SCW-related *in house* transcriptome dataset came from the *Arabidopsis* mutant, *wat1 (walls are thin 1)*, which has little to no SCW in fibers (Ranocha et al., 2010). In this mutant, the transcript levels of many genes associated with the regulation and/or the biosynthesis of the different SCW wall polymers were dramatically reduced in keeping with the mutant phenotype. Within the genes up/or down-regulated in the mutant background, we identified 97 TFs including some well-known SCW-regulating TFs such as *SND1*, *SNT1*, and *MYB46* (Table S1). The second transcriptome dataset was comprised of 240 TFs (Table S2) that exhibited de-regulated expression in *Arabidopsis* lines over-expressed the SCW-master activator *EgMYB2*, which is a *Eucalyptus* R2R3 MYB TF highly expressing in xylem cells undergoing SCW thickening (Goicoechea et al., 2005). EgMYB2 is able to activate the promoters of lignin (Goicoechea et al., 2005), cellulose, and xylan biosynthetic genes (Zhong and Ye, 2009), leading to thicker SCW in *EgMYB2* over-expressing lines in tobacco (Goicoechea et al., 2005; De Micco et al., 2012). Moreover, the closest orthologs of *EgMYB2* in *Arabidopsis AtMYB46* and *AtMYB83* encode for master regulators capable of activating the whole SCW biosynthetic program (Zhong et al., 2007a; McCarthy et al., 2009), and EgMYB2 was able to complement the *myb46-myb83* double mutant (Zhong et al., 2010). The third transcriptome dataset included 309 TFs (Table S3) deregulated in *Arabidopsis* lines over-expressing the SCW-repressor, *EgMYB1* (Legay et al., 2010). *EgMYB1* over-expressors exhibited fewer lignified fibers particularly in the interfascicular zones and reduced SCW thickenings. Klason lignin content was moderately but significantly reduced and decreased transcript accumulation was observed for genes involved in the biosynthesis of lignins, cellulose, and xylan (Legay et al., 2010). Finally, the fourth dataset was composed of 87 TFs (Table S4) that were the *Arabidopsis* orthologs of *Eucalyptus* TFs preferentially expressed in differentiating xylem (Rengel et al., 2009), a tissue that is particularly rich in cells undergoing SCW deposition and lignification. Altogether, these four transcriptomic datasets allowed us to identify a total of 502 candidate TFs. To narrow down the number of candidates for functional validation, we selected 186 that were identified in two datasets (Table S5). It should be noted that 43 of those were found in three data sets and only three were common to the four datasets *bHLH5* (AT5G46760), *IAA9* (AT5G65670), and *AP2 TF RAP2.2* (AT3G14230).

### Cross-comparison with publicly available microarray data

To further narrow down the selection of the 186 candidate TFs for further functional analysis, we examined their *in silico* expression patterns using Genevestigator (Hruz et al., 2008). We restricted our list to genes that were preferentially and/or highly expressed in situations in which SCW formation is prevalent i.e., in xylem, the basal part of the inflorescence stem, and/or in cell suspension cultures undergoing *in vitro* SCW formation (Kubo et al., 2005). This *in silico* expression screen allowed us to obtain a final list of 80 candidate SCW TFs (Table 1).

**Table 1:** **List of 80 candidate TFs obtained after cross-comparison of transcriptomic datasets**.

**ID**	**Annotation**	***in house* data sets (Log_2_Ratio)**				**Publicly available data**		**No. of ordered mutants**
		**wat1**	**OE EgMYB2**	**OE EgMYB1**	**Eg Xylem**	TE induction 6d^*^	Xylem^**^		
AT3G50260	AP2 TF DREB, CEJ1 (subfamily A-5)	2.7	−1.6	−2.44		0.3	Low_ns	0
AT5G61590	AP2 TF ERF (subfamily B-3)	3.4	−1.1	2.1		3.7	High_p	1
AT5G07580	AP2 TF ERF (subfamily B-3)		2.2	2.4		0.9	High_p	1
AT5G51190	AP2 TF ERF (subfamily B-3)	−3.2	1.8	2.4		1.5	High_p	1
AT5G13330	AP2 TF ERF (subfamily B-4)	7.3	−0.6			1.4	High_p	0
AT3G14230	AP2 TF ERF RAP2.2 (subfamily B-2)	2.0	1.4	1.1	x	0.2	High_p	2
AT1G43160	AP2 TF ERF RAP2.6 (subfamily B-4)	2.2	−0.7	−1.5		−0.2	Low_ns	0
AT1G68840	AP2 TF ERF RAP2.8 (RAV2)	1.9	−2.0	−2.3		−2.0	Medium_ns	0
AT3G25890	AP2 TF ERF, CRF11 (subfamily B-6)	2.0	1.0	1.2		0.1	Medium_p	0
AT5G25190	AP2 TF ERF, ESE3 (subfamily B-6)	−2.3			x	2.0	Low_ns	0
AT2G44840	AP2 TF ERF13 (subfamily B-3)	−2.8		−0.8	x	0.0	Medium_u	1
AT5G47230	AP2 TF ERF5 (subfamily B-3)	−2.7	1.3			−0.4	Medium_u	0
AT5G60450	ARF4	−1.7	2.0	1.9		−0.4	High_s	2
AT1G30330	ARF6		1.3	1.6	x	−0.8	Medium_u	1
AT5G54680	bHLH105	−3.7	0.6		x	−0.3	High_u	1
AT2G46510	bHLH17		1.1		x	−0.2	Medium_u	0
AT1G68810	bHLH30	−2.4	2.0	1.8		2.4	High_s	3
AT3G25710	bHLH32	−2.1	1.2	1.6		0.0	High_s	2
AT5G46760	bHLH5	1.8	1.5	1.2	x	0.7	High_p	2
AT1G75390	bZIP TF	−2.1	0.6			−2.9	High_p	0
AT2G18160	bZIP TF	−1.8	0.9			2.4	High_p	1
AT3G51960	bZIP TF	2.3	−0.8	−0.9		0.5	Low_u	0
AT3G56850	bZIP TF (ABA_REB3)		0.6	0.6	x	0.8	Medium_u	0
AT1G72830	CCAAT-binding TF	2.8	1.6	1.5		−0.6	High_s	0
AT3G28730	HMG		1.1	0.7	x	−0.4	High_p	0
**AT2G34710**	**homeobox-leucine zipper TF (HB14)**			0.8	x	4.1	High_p	0
**AT1G52150**	**homeobox-leucine zipper TF (HB15)**		1.3		x	4.2	High_s	3
AT5G65310	homeobox-leucine zipper TF (HB5)	−2.1		0.9		−2.5	Low_u	3
AT5G41410	homeodomain TF (BEL1)	2.2	−1.2	−0.6		3.0	Low_ns	0
AT5G02030	homeodomain TF (BELLRINGER)		1.4	1.6		0.2	High_p	0
AT4G34610	homeodomain TF (BLH6)	−2.0	0.7	0.7		1.4	Low_p	2
AT1G70510	homeodomain TF (KNAT2)		1.4	2.1		−0.1	Medium_s	0
**AT1G62990**	**homeodomain TF (KNAT7)**	−7.9	2.8	2.4		2.6	High_s	0
AT1G62360	homeodomain TF (SHOOT MERISTEMLESS)		3.9	3.7	x	−0.1	High_s	0
AT4G28640	IAA11	−2.6	1.6	1.8		1.4	Medium_s	1
AT2G33310	IAA13		2.3	1.7	x	1.1	High_p	0
AT3G04730	IAA16		1.7	1.4		1.2	High_u	0
AT2G46990	IAA20		1.8	1.5		−0.3	Low_u	0
AT5G25890	IAA28	1.4	2.3	2.1		3.3	High_s	1
AT2G22670	IAA8		1.2	1.3		0.6	High_p	0
AT5G65670	IAA9	−1.8	0.9	1.2	x	0.8	High_p	0
AT1G10200	LIM TF			0.7	x	1.4	Medium_u	0
AT1G01060	myb related TF (LHY)	−1.8	2.6		x	0.4	High_u	1
**AT1G63910**	**MYB103**	−11.8		2.4		3.2	Medium_s	1
AT1G48000	MYB112	2.8	−1.7	−1.5		−0.1	Low_u	0
**AT1G66230**	**MYB20**	−3.1				1.0	Medium_u	1
AT5G07690	MYB29	−4.4	1.9	2.3		−0.2	Low_u	0
AT4G38620	MYB4		1.4		x	−1.3	Medium_p	3
**AT4G12350**	**MYB42**		1.3	1.0		0.6	Low_u	0
**AT5G12870**	**MYB46**	−2.8	0.9	1.4		5.3	Medium_s	2
AT3G46130	MYB48	3.0	1.4	1.8		0.1	High_s	0
**AT1G17950**	**MYB52**	−2.1	2.3	2.4		3.3	Medium_s	2
AT5G59780	MYB59	4.7	1.0	0.8		2.7	High_p	1
AT1G09540	MYB61	−2.4		1.8		no probe	no probe	0
**AT1G79180**	**MYB63**	−5.9	1.3			2.3	Medium_s	0
**AT2.36650**	**MYB75**		−1.3	−1.9		0.1	Low_u	0
AT3G50060	MYB77	−3.2	1.4	0.8		−0.5	Medium_u	0
**AT4G22680**	**MYB85**	−2.1	2.2	1.6		2.1	Low_u	1
AT5G05790	myb-like TF	−1.9	0.7	0.9		1.7	High_s	0
AT5G17300	myb-like TF	−1.8	0.8			1.2	Medium_u	0
AT3G11280	myb-like TF	1.9		0.6		4.5	High_p	3
AT1G12260	NAC007, VND4	−1.9				5.0	Low_u	1
**AT1G32770**	**NAC012, SND1, NST3**	−2.6		1.8		0.0	Medium_s	0
**AT2G46770**	**NAC043, NST1**	−0.8			x	no probe	no probe	0
**AT5G64530**	**NAC104, XND1**			0.9	x	3.6	High_s	0
**AT5G13180**	**NAC83, VND-INTERACTING2 (VNI2)**		−0.7		x	3.3	High_u	0
AT4G37750	AINTEGUMENTA (ANT)	−5.4		1.0		0.1	Low_p	0
AT1G21450	scarecrow-like TF 1 (SCL1)		1.2		x	2.2	High_p	1
AT2G47070	squamosa TF-like 1 (SPL1)		0.8		x	0.7	High_u	0
**AT2G44745**	**WRKY12**	−2.9	2.3	2.7		−0.1	High_s	1
AT2G30250	WRKY25	2.6	−0.7	−0.6		−1.2	Medium_u	2
AT4G23550	WRKY29	−7.7	2.1	1.6		no probe	no probe	1
AT1G80840	WRKY40	−3.0			x	−0.4	High_u	1
AT3G01970	WRKY45	3.4	−0.8	−1.2		−0.5	Medium_u	0
AT5G13080	WRKY75	3.5	−0.8	−0.9		−0.3	Low_ns	0
AT2G28510	zinc finger Dof-type	−1.7	−0.8	−0.9		0.2	Low_ns	0
AT1G68360	zinc finger TF	−3.7	1.8	2.0		1.1	High_s	0
AT2G40140	zinc finger TF (CCCH-type)	−1.8	0.6			0.3	High_u	0
AT3G55980	zinc finger TF (CCCH-type)	−3.6			x	0.5	High_u	2
AT3G46620	zinc finger TF-n129	−3.4				1.6	High_u	2

* *TE induction 6d, 6 days after induction (1 uM brassinolide and 10 mM H3BO3) when tracheary element were actively forming*.

** *Xylem in hypocotyl of adult plant; High, Medium, and Low for their expression level; _s, specific expression in xylem; _p, preferential expression; _u, ubiquitous expression; _ns, higher expression in non-xylem cells. Bold indicates known genotypes, and corresponding secondary wall or xylem cell identity phenotypes*.

**Figure 2:**
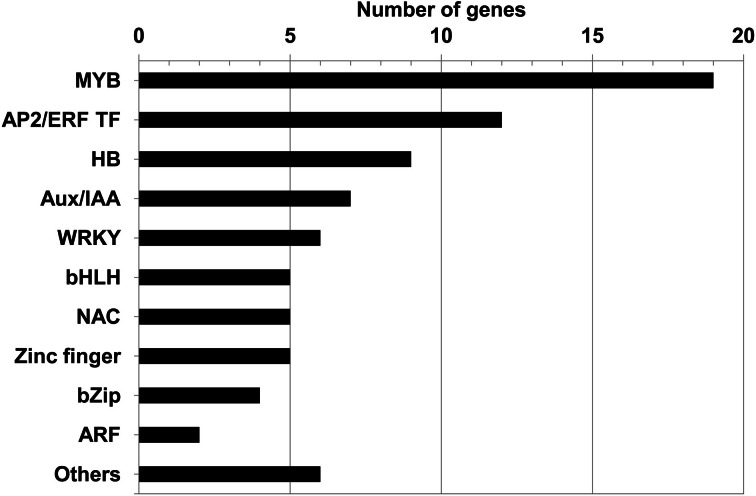
**Distribution of the 80 SCW candidate TFs according to family.** Amongst them, four families have been shown previously to regulate SCW formation: MYB, NAC, HB (Homeodomain containing TF), and WRKY; three TF families are involved in hormone signaling including ARF, Aux/IAA, and AP2 ERF TF families. “Others” include the following TF families: LIM, CCAAT binding, HMG (High Mobility Group, belonging to a transcription complex), AINTEGUMENTA (ANT; AP2 like TF), Scarecrow-like TF1 (SCL1), and Squamosa-like1 (SPL1).

The most represented families in decreasing order were the MYB TF family (containing 19 members), the AP2 ERF (Ethylene Response Factor) TF family (12), the HB (HomeoBox protein) TF family (9), and the Aux/IAA family (7) (Figure 2). The other TFs belonged to the WRKY (5), NAC (5), bHLH (5), Zinc finger TF (5), bZip (3), and ARF (2) families, respectively.

It is noteworthy that 16 of the 80 candidates were already shown to regulate SCW formation. They include eight MYB TF (*MYB20*, *MYB42*, *MYB46*, *MYB52*, *MYB61*, *MYB63*, *MYB75*, *MYB85*, and *MYB103*) (Zhong et al., 2007a, 2008; Ko et al., 2009; Zhou et al., 2009; Ohman et al., 2013), four NAC TF (*SND1*, *NST1 XND1*, and *VNI2*) (Mitsuda et al., 2005; Zhong et al., 2006; Zhao et al., 2008; Yamaguchi et al., 2010), three Homeodomain containing TF (*HB14*, *HB15*, and KNAT7) (McConnell et al., 2001; Emery et al., 2003; Kim et al., 2005; Zhong et al., 2008) and one WRKY TF (*WRKY12*) (Wang et al., 2010) (complete list in Table 1). This significant proportion of characterized SCW related TF in our final list validates well the strategy used in this study. For example, *MYB46* (Zhong et al., 2007a), *MYB85* (Zhong et al., 2008), and *WRKY12* (Wang et al., 2010) were present in three of the four transcriptomic datasets.

### Phenotypes of TF T-DNA mutant or RNAi transgenic lines

**Figure 3:**
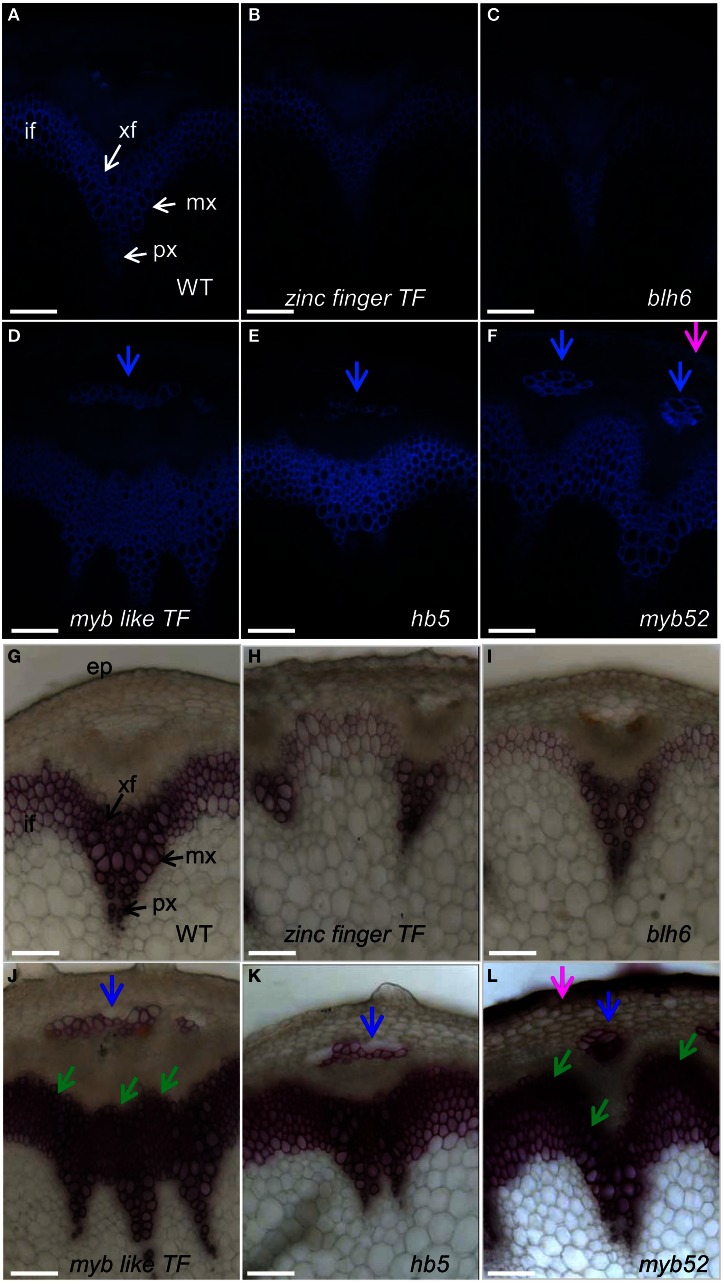
**Stem cross sections of five T-DNA mutant lines presenting either hypo or hyperlignified SCWs.** Sections of wild-type plant and T-DNA mutants were observed under UV light **(A–F)** or stained with phloroglucinol-HCl **(G–L)**. Phloem cap cells and ectopic lignification in epidermal cells are indicated by blue and pink arrows, respectively. Observations were made at the basal part of inflorescence stems at the stage of newly formed green siliques, about two weeks after bolting, when the inflorescence stems reached 20 cm height. if, interfascicular fiber; xf, xylary fiber; mx, metaxylem; px, protoxylem; sx, secondary xylem; ep, epidermis. Scale bar: 20 μm.

**Figure 4:**
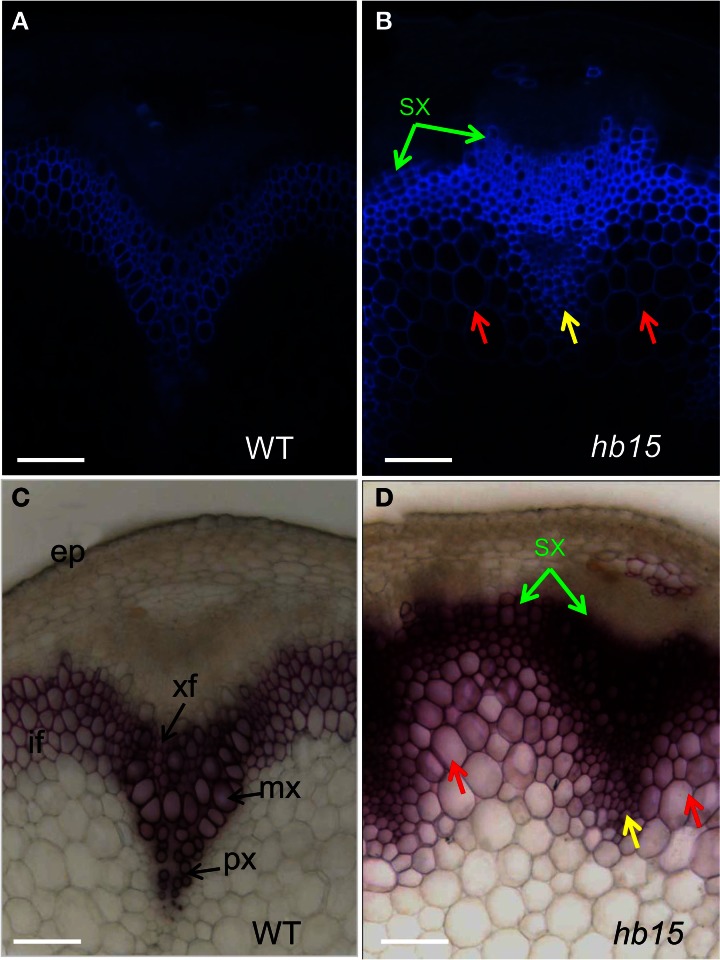
**Stem sections of the *hb15* T-DNA mutant.** Auto-fluorescence observed under UV light of wild type **(A)** and *hb15* mutant **(B)** Phloroglucinol-HCl staining of lignin in wild type **(C)** and *hb15* mutant **(D)**. Ectopic lignification in large parenchyma cells and in small parenchyma cells surrounding protoxylem is indicated by red arrows and yellow arrows, respectively; precocious secondary walled secondary xylem formation is indicated by green arrow. if, interfascicular fiber; xf, xylary fiber; mx, metaxylem; px, protoxylem; sx, secondary xylem; ep, epidermis. Scale bar: 20 μm.

We then collected and characterized publicly available T-DNA mutant lines or RNAi transgenic lines that corresponded to 43 of the 80 candidate genes (Table S6). The information concerning the different lines including T-DNA insertion position and in-house databases source is presented in Table S6. Phenotyping was performed on 20 cm-high mutant stems grown in short-day growth conditions. Under these conditions, the basal part of the stem abundantly develops cells undergoing SCW thickening (xylem vessel cells, xylary fiber cells, and interfascicular fiber cells). Histological analyses of SCW were performed using the natural auto fluorescence of phenolic compounds under UV-light as well as phloroglucinol-HCl staining, which is indicative of the lignin content. We found significant alteration of lignin profiles in six mutant lines corresponding to two MYB TFs: *MYB like TF* (AT3G11280) and *MYB52* (AT1G17950), three HomeoBox TF *HB5* (AT5G65310), *BLH6* (AT4G34610), and *HB15* (AT1G52150) and a *Zinc finger TF* (AT3G46620), although the overall organization of vascular bundles and interfascicular fibers was not altered in these six mutant lines (Figures 3, 4).

Under UV-light the intensity of auto-fluorescence was lower in *zinc finger TF* (Figure 3B) and in *blh6* (Figure 3C) mutant lines in both vascular bundles and interfascicular regions as compared to the control (Figure 3A), suggesting a global decrease in phenolic compound deposition. The SCW in xylem vessels, xylary fibers, and interfascicular fibers were observed in more detail using phloroglucinol-HCl staining. Little to no SCW was deposited in xylary fibers (Figures 3H,I) and moreover, SCW thickness was largely reduced in interfascicular fibers (thin and weak phloroglucinol staining) as compared to wild-type (Figure 3G) suggesting that these lines were hypolignified.

Auto-fluorescence under UV light was more intense in *myb like TF* (Figure 3D*)*, *hb5* (Figure 3E), and *myb52* (Figure 3F) lines than in controls (Figure 3A), especially in the interfascicular region, suggesting an increased deposition of phenolic compounds and possibly lignins. This was further confirmed by a massive and intense phloroglucinol staining indicating an enhanced lignin deposition in the interfascicular fiber and xylary fiber cells of these mutants (Figures 3J–L). Extra-layers of cells with lignified SCW were detected in the external layers of both interfascicular fibers and metaxylem vessels in two lines *myb-like TF* (Figure 3J, green arrows) and *myb52* (Figure 3L, green arrows) as compared to the control (Figure 3A). This observation suggests that secondary xylem formation was enhanced and appeared earlier than in wild-type. A strong fluorescent signal was also detected in the phloem cap cells (Figures 3D–F, blue arrows) in all three highly auto-fluorescent lines suggesting a transition of phloem cap cells to phloem sclereids (highly lignified) which was further confirmed by phloroglucinol staining (Figures 3J–L, blue arrows). Similarly, auto-fluorescent signals (Figure 3F) and strong phloroglucinol-HCl staining (Figure 3L, pink arrow) were detected in the epidermal cells of some *myb52* T-DNA insertion lines revealing an ectopic deposition of lignin.

Both auto-fluorescence and phloroglucinol staining of stem sections of *hb15* (Figures 4B,D) showed that large parenchyma cells adjacent to the inner side of the interfascicular fibers (red arrow), as well as smaller xylem parenchyma cells surrounding the protoxylem (yellow arrow) exhibited lignified SCW. The corresponding cells in the control have non-lignified primary walls (Figures 4A,C). As compared to the control, extra layers of cells with lignified SCW were present in the most external rows of the interfascicular fibers and xylem (Figures 4B,D, green arrows) suggesting an enhanced and early formation of secondary xylem. Moreover, both xylary and interfascicular fibers in *hb15* lines exhibited both a more intense auto-fluorescence and staining by phloroglucinol than that of the control suggesting higher lignin content.

**Figure 5:**
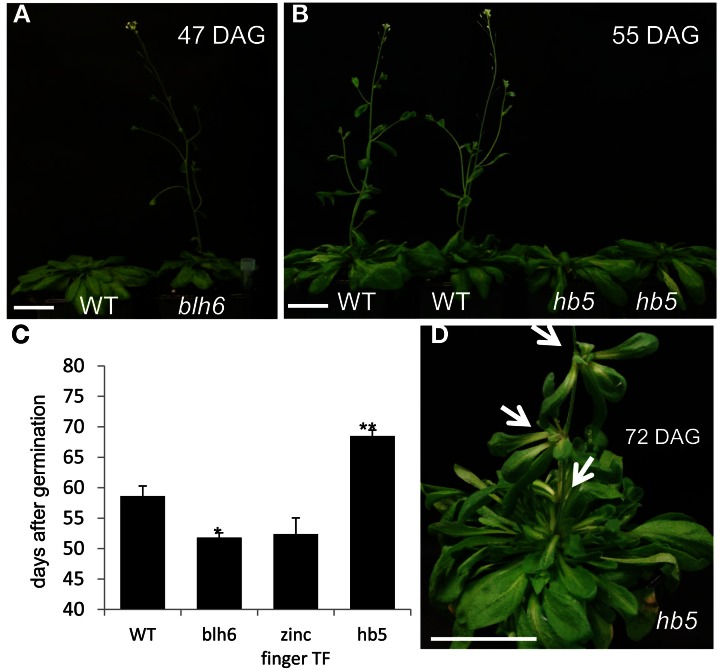
**Comparison of flowering time between wild-type and mutants plants. (A)** Early flowering hypo-lignified line *blh6*. **(B)** Delayed flowering of hyperlignified line *bh5*. **(C)** Flowering time comparison between wild type and hypo or hyperlignified mutants. Significant and very significant statistical differences to wild type are represented by ^*^ or ^**^, respectively (Student *t*-test). **(D)** Aerial rosette formation on the inflorescence of *hb5* mutants. Scale bar: 5 cm. DAG, days after germination. Arrows point to aerial rosettes.

The overall growth behavior of the mutants did not differ significantly from the controls, except the bolting and flowering time were altered in three of the mutant lines. Hypolignified *blh6* and *zinc finger* lines bolted and flowered earlier than controls (Figures 5A,C) whereas the hyperlignified *hb15* line exhibited delayed bolting and flowering (Figures 5B,C). In addition, *hb15* mutants exhibited aerial rosettes at the base of the lateral inflorescence branches instead of growing cauline leaves as in wild-type plants (Figure 5D).

### Co-expression analysis of candidate TFs genes

Since it is known that transcriptionally coordinated genes tend to be functionally related (Ruprecht and Persson, 2012), we performed co-expression analyses for the six candidate genes in order to further validate their role in controlling SCW synthesis and get some clues about their function. The co-expression genes lists were generated using the Genevestigator platform (https://www.genevestigator.com), *Arabidopsis* co-expression data mining tools and GeneCAT. All six candidate TFs were co-expressed with genes related to cell wall formation (Tables 2, 3 and Tables S7–S10) albeit to different extents ranging from 10 to 66% of SCW associated genes amongst the 50 first co-expressed genes. The most remarkably high co-expression profiles were found for *MYB52 and BLH6*.

**Table 2:** **Top 50 co-expressed genes with *BLH6* using Genevestigator platform**.

**Probeset**	**AGI**	**Gene symbol**	**Score**	**Description**
253247_at	AT4G34610	BLH6	1	BEL1-like homeodomain 6
**252025_at**	**AT3G52900**		**0.75**	Family of unknown function (DUF662)^*^
250600_at	AT5G07800		0.73	Flavin-binding monooxygenase family protein
**264573_at**	**AT1G05310**		**0.71**	**Pectin lyase-like superfamily protein**
245657_at	AT1G56720		0.71	Protein kinase superfamily protein
**251069_at**	**AT5G01930**	**MAN6**	**0.70**	**Glycosyl hydrolase superfamily protein**
263629_at	AT2G04850		0.70	Auxin-responsive family protein
**250933_at**	**AT5G03170**	**FLA11**	**0.69**	**FASCICLIN-like arabinogalactan-protein 11**
**262796_at**	**AT1G20850**	**XCP2**	**0.68**	**xylem cysteine peptidase 2**
245864_at	AT1G58070		0.68	Unknown protein
258938_at	AT3G10080		0.68	RmlC-like cupins superfamily protein
**251131_at**	**AT5G01190**	**LAC10**	**0.68**	laccase 10^*^
**246827_at**	**AT5G26330**		**0.67**	Cupredoxin superfamily protein^*^
246439_at	AT5G17600		0.67	RING/U-box superfamily protein
251249_at	AT3G62160		0.66	HXXXD-type acyl-transferase family protein
**257757_at**	**AT3G18660**	**PGSIP1,GUX1**	**0.66**	**Glucuronic acid substitution of xylan 1**
**264184_at**	**AT1G54790**		**0.65**	GDSL-like Lipase/Acylhydrolase family protein^*^
253876_at	AT4G27430	CIP7	0.65	COP1-interacting protein 7
259688_at	AT1G63120	RBL2	0.65	RHOMBOID-like 2
**267094_at**	**AT2G38080**	**LAC4,IRX12,LMCO4**	**0.65**	**Laccase/Diphenol oxidase family protein**
250664_at	AT5G07080		0.64	HXXXD-type acyl-transferase family protein
**250322_at**	**AT5G12870**	**MYB46**	**0.64**	**myb domain protein 46**
250120_at	AT5G16490	RIC4	0.64	ROP-interactive CRIB motif-containing protein 4
251297_at	AT3G62020	GLP10	0.64	germin-like protein 10
256367_at	AT1G66810		0.64	Zinc finger C-x8-C-x5-C-x3-H type family protein
265277_at	AT2G28410		0.63	Unknown protein
**246425_at**	**AT5G17420**	**IRX3,MUR10,CESA7**	**0.63**	**Cellulose synthase family protein**
266424_at	AT2G41330		0.62	Glutaredoxin family protein
**253798_at**	**AT4G28500**	**ANAC073,SND2**	**0.62**	**NAC domain containing protein 73**
**260867_at**	**AT1G43790**	**TED6**	**0.62**	**Tracheary element differentiation-related 6**
251050_at	AT5G02440		0.61	Unknown protein
**264493_at**	**AT1G27440**	**GUT1,GUT2,IRX10**	**0.61**	**Exostosin family protein**
261653_at	AT1G01900	SBTI1.1	0.61	Subtilase family protein
262922_at	AT1G79420		0.61	Protein of unknown function (DUF620)
**266244_at**	**AT2G27740**		**0.61**	Family of unknown function (DUF662)^*^
**257896_at**	**AT3G16920**	**CTL2**	**0.60**	**chitinase-like protein 2**
**249070_at**	**AT5G44030**	**IRX5,NWS2,CESA4**	**0.60**	**cellulose synthase A4**
**247590_at**	**AT5G60720**		**0.60**	Protein of unknown function, DUF547^*^
**267037_at**	**AT2G38320**	**TBL34**	**0.60**	TRICHOME BIREFRINGENCE-LIKE 34^*^
**247648_at**	**AT5G60020**	**LAC17**	**0.60**	**laccase 17**
**257151_at**	**AT3G27200**		**0.60**	Cupredoxin superfamily protein^*^
**256155_at**	**AT3G08500**	**MYB83**	**0.60**	**myb domain protein 83**
**254618_at**	**AT4G18780**	**IRX1,CESA8,LEW2**	**0.60**	**Cellulose synthase family protein**
**251630_at**	**AT3G57420**		**0.60**	Protein of unknown function (DUF288)^**^
**260914_at**	**AT1G02640**	**BXL2**	**0.60**	beta-xylosidase 2^**^
258357_at	AT3G14350	SRF7	0.59	STRUBBELIG-receptor family 7
**263841_at**	**AT2G36870**	**XTH32**	**0.59**	**xyloglucan endotransglucosylase/hydrolase 32**
259657_at	AT1G55180	PLDEPSILON,PLDALPHA4	0.59	phospholipase D alpha 4
**255150_at**	**AT4G08160**		**0.59**	xylanase, glycosyl hydrolase family 10 protein^*^
**246512_at**	**AT5G15630**	**IRX6,COBL4**	**0.58**	**COBRA-like extracellular glycosyl-phosphatidyl inositol-anchored protein family**
**253191_at**	**AT4G35350**	**XCP1**	**0.58**	**xylem cysteine peptidase 1**

* *Annotated involved in “xylan biosynthetic process” and/or “cell wall biogenesis” and/or “cell wall macromolecule metabolic process” in Tair (http://www.arabidopsis.org)*.

** *Located in cell wall according to Tair. Bold indicates related to cell wall formation*.

**Table 3:** **Top 50 co-expressed genes with *MYB52* using Genevestigator platform**.

**Probeset**	**AGI**	**Gene symbol**	**Score**	**Description**
255903_at	AT1G17950	MYB52,BW52	1	myb domain protein 52
**245735_at**	**AT1G73410**	**MYB54**	**0.90**	**myb domain protein 54**
252550_at	AT3G45870		0.82	nodulin MtN21 /EamA-like transporter family protein
**264559_at**	**AT1G09610**	**GXM3**	**0.82**	**GLUCURONOXYLAN METHYLTRANSFERASE 3 (DUF579)**
248761_at	AT5G47635		0.81	Pollen Ole e 1 allergen and extensin family protein
**252211_at**	**AT3G50220**	**IRX15**	**0.81**	**Protein of unknown function (DUF579)**
**266783_at**	**AT2G29130**	**LAC2**	**0.80**	laccase 2^*^
**257233_at**	**AT3G15050**	**IQD10**	**0.79**	IQ-domain 10^*^
256054_at	AT1G07120		0.79	Tetratricopeptide repeat (TPR)-like superfamily protein
**254277_at**	**AT4G22680**	**MYB85**	**0.78**	**myb domain protein 85**
**264493_at**	**AT1G27440**	**GUT1,GUT2,IRX10**	**0.77**	**Exostosin family protein**
**250770_at**	**AT5G05390**	**LAC12**	**0.77**	laccase 12^*^
246425_at	**AT5G17420**	**IRX3,MUR10,CESA7**	**0.76**	**Cellulose synthase family protein**
251009_at	AT5G02640		0.76	Unknown protein
259584_at	AT1G28080		0.76	RING finger protein
**248121_at**	**AT5G54690**	**GAUT12,IRX8,LGT6**	**0.75**	**galacturonosyltransferase 12**
261928_at	AT1G22480		0.74	Cupredoxin superfamily protein
254170_at	AT4G24430		0.74	Rhamnogalacturonate lyase family protein
**246512_at**	**AT5G15630**	**IRX6,COBL4**	**0.74**	**COBRA-like4**
264495_at	AT1G27380	RIC2	0.74	ROP-interactive CRIB motif-containing protein 2
245105_at	AT2G41610		0.74	Unknown protein
**253327_at**	**AT4G33450**	**MYB69**	**0.74**	**myb domain protein 69**
**257896_at**	**AT3G16920**	**CTL2**	**0.73**	**chitinase-like protein 2**
**255150_at**	**AT4G08160**		**0.73**	glycosyl hydrolase family 10 protein^*^
266714_at			0.73	No_match
**260326_at**	**AT1G63910**	**MYB103**	**0.73**	**myb domain protein 103**
**248907_at**	**AT5G46340**	**RWA1**	**0.72**	**REDUCED WALL ACETYLATION 1, O-acetyltransferase family**
**266488_at**	**AT2G47670**		**0.72**	**Plant invertase/pectin methylesterase inhibitor family protein**
**253379_at**	**AT4G33330**	**PGSIP3,GUX2**	**0.72**	**plant glycogenin-like starch initiation protein 3**
**261399_at**	**AT1G79620**		**0.72**	Leucine-rich repeat protein kinase family protein^*^
**251478_at**	**AT3G59690**	**IQD13**	**0.71**	IQ-domain 13^*^
**265463_at**	**AT2G37090**	**IRX9**	**0.71**	**GT43 glycosyl transferase43**
**266708_at**	**AT2G03200**		**0.71**	Eukaryotic aspartyl protease family protein^*^
248887_at	AT5G46115		0.71	Unknown protein
267414_at	AT2G34790	MEE23,EDA28	0.70	FAD-binding Berberine family protein
246344_at	AT3G56730		0.70	Putative endonuclease or glycosyl hydrolase
**263470_at**	**AT2G31900**	**ATMYO5,XIF**	**0.70**	myosin-like protein XIF^*^
264305_at	AT1G78815	LSH7	0.70	LIGHT SENSITIVE HYPOCOTYLS 7, Protein of unknown function (DUF640)
**251131_at**	**AT5G01190**	**LAC10**	**0.69**	laccase 10^*^
**251093_at**	**AT5G01360**	**TBL3**	**0.69**	Plant protein of unknown function (DUF828)^*^
**247264_at**	**AT5G64530**	**XND1,ANAC104**	**0.69**	**xylem NAC domain 1**
260430_at	AT1G68200		0.69	Zinc finger C-x8-C-x5-C-x3-H type family protein
**261809_at**	**AT1G08340**		**0.69**	Rho GTPase activating protein^*^
**253798_at**	**AT4G28500**	**SND2,NAC073**	**0.69**	**NAC domain containing protein 73**
246342_at	AT3G56700	FAR6	0.69	Fatty acid reductase 6
**247030_at**	**AT5G67210**	**IRX15-L**	**0.69**	**Protein of unknown function (DUF579)**
**254618_at**	**AT4G18780**	**IRX1,CESA8,LEW2**	**0.69**	**Cellulose synthase family protein**
**247590_at**	**AT5G60720**		**0.68**	Protein of unknown function, DUF547^*^
**249439_at**	**AT5G40020**		**0.68**	Pathogenesis-related thaumatin superfamily protein^*^
**253877_at**	**AT4G27435**		**0.68**	Protein of unknown function (DUF1218)^*^
253710_at	AT4G29230	NAC075	0.68	NAC domain containing protein 75

* *Annotated involved in “xylan biosynthetic process” and/or “cell wall biogenesis” and/or “cell wall macromolecule metabolic process” in Tair (http://www.arabidopsis.org). Bold indicates related to cell wall formation*.

Among the top 50 genes co-expressed with *BLH6*, 30 (60%) were related to SCW formation (Table 2). Notably, they included genes involved in the biosynthesis of the three main cell wall polymers i.e., three main SCW cellulose synthases genes (CESAs) (*IRX5/CESA4*, *IRX3/CESA7*, and *IRX1/CESA8)*, three genes implicated in xylan biosynthesis [*IRX10/GUT2*, *GUX1* (Glucuronic acid substitution of xylan1), and *XTH32* (xyloglucan endotransglucosylase/hydrolase 32) (Brown et al., 2009; MacMillan et al., 2010)] as well as two laccase genes (*IRX12/LAC4* and *LAC17)* involved in monolignol polymerization (Berthet et al., 2011). Moreover, several SCW master transcriptional regulators were also co-expressed with *BLH6*, such as *MYB46*, *MYB83*, and *SND2* (Zhong et al., 2008; Zhong and Ye, 2012).

The co-expression analysis *of MYB52* revealed that 33 of the top 50 genes (66%) co-expressed with *MYB52* were related to SCW formation. The list of co-expressed genes included genes encoding key biosynthetic enzymes of the main SCW polymers i.e., cellulose (*IRX3/CESA7*, *IRX1/CES8*, *IRX6/COBL4*, and *CTL2*), hemicelluloses (*IRX10/GUT1*, *IRX8*, *IRX9*, *IRX15*, and *IRX15-L*), and possibly lignins (*LAC2* and *LAC10*). *MYB52* was also co-expressed with many SCW regulators such as *MYB54*, *MYB85*, *MYB69*, *MYB103*, *SND2/NAC073*, and *XND1/NAC104* (Xylem NAC Domain1).

## Discussion

Functional genomics approaches developed during the last decade have generated numerous candidate genes related to SCW formation in *Arabidopsis* and other plant species. Whereas these large individual gene lists make difficult the choice of the most promising candidates for the further functional validation, meta-analyses combining multiple transcriptomic data sets offer a new way to reveal some core regulators.

By cross-comparing four SCW-related transcriptomic datasets, we selected 186 TFs present in at least two experimental conditions. Since these datasets came from very different backgrounds (mutants and over-expressors of SCW regulatory genes as well as orthologs of *Eucalyptus* xylem expressed genes), the selection of genes appearing in more than one dataset likely helped us to identify “core regulators” of SCW formation but might also have filtered out some more specific regulators. We further restricted the candidate gene list by including *in silico* analyses of their expression making the hypothesis that TFs expressed highly or preferentially in xylem tissues and/or during tracheary elements formation would be the most promising candidates. Indeed this strategy was successful since among the 80 genes that came out, 16 have already been reported to be regulators of the SCW. They included, for instance, the master regulators *SND1* and *MYB46* as well as the lignin-specific *MYB85*.

Forty-three mutant lines were phenotyped but only six exhibited a notable cell wall phenotype. This high proportion of mutants without phenotype is not surprising since many mutants targeting only one TF are known to yield mild to no phenotype (Okushima et al., 2005; Overvoorde et al., 2005; Jensen et al., 2011; Ruprecht et al., 2011). This is particularly true for multigene families TFs where functional redundancy prevents the observation of distinct phenotypes in knock-out mutants. This is indeed the case for a large proportion of the SCW regulators characterized hitherto including some of the sixteen highlighted here. For instance, whereas a single mutant of the SCW master transcriptional activator *MYB46* did not exhibit any cell wall phenotype, the double knock out mutant *myb46/myb83* with its closest ortholog *MYB83* showed a severe reduction of SCW thickness (Zhong et al., 2007a; McCarthy et al., 2009). Therefore, genes for which the corresponding single mutants exhibited no phenotype in this study may still be interesting candidates taking part in the regulation of SCW formation. Further experiments using over-expressors and/or mutants of two or more paralog genes would increase the probability of obtaining informative phenotypes and insight into their functions. Our *in silico* analyses pointed out some very promising genes which should be further characterized using such approaches.

The most abundantly represented TF family in our list was the MYB family (19 members) of which eight (belonging to the R2R3 subfamily) have already been shown to regulate either the phenylpropanoid pathway and/or the SCW formation. It is the case for MYB46 (Zhong et al., 2007a), MYB63 (Zhou et al., 2009), MYB85 (Zhong et al., 2008), and MYB103 (Ohman et al., 2013). We phenotyped *myb52* insertion lines that exhibited a strong hyperlignification phenotype, thus suggesting that MYB52 could be a repressor of the lignin biosynthesis and possibly of the whole SCW formation. This result is in apparent contradiction with a previous study showing that the dominant repression of *MYB52* caused a severe reduction in SCW thickening in both interfascicular fibers and xylary fibers of the inflorescence stem (Zhong et al., 2008). The authors concluded that *MYB52* was an activator of the SCW although no phenotype was detectable when over-expressed. A likely explanation to these apparent discrepancies is that *MYB52* encodes a transcriptional repressor as clearly suggested by our knock-out mutant phenotype and therefore its dominant repression would result in a stronger transcriptional repression. *MYB52* appeared to be tightly co-expressed with *MYB54* and *WAT1*. It is also co-expressed with several cellulose and xylan biosynthetic genes and with MYB85, a specific regulator of the lignin biosynthesis (Zhong et al., 2008). Altogether, these results suggest for MYB52 a repressor role of the whole SCW program although this needs to be supported by further experiments.

Besides these canonic R2R3 MYBs, four MYB-like proteins were present in the candidate list and one mutant was analyzed. Although none of these MYB-like factors has been yet reported as regulators of the SCW, the *myb-like TF* T-DNA mutant had a clear hyperlignification phenotype suggesting a repressor role of the lignin biosynthesis and/or SCW. The *myb-like TF* gene was annotated in TAIR (http://www.arabidopsis.org) as a putative MYB domain containing TF able to interact with the gene product of vacuolar ATPase subunit B1 (VHA-B1). Interestingly, it is highly co-expressed with a newly reported gene *XIP1* (*XYLEM INTERMIXED WITH PHLOEM1*), a leucine-rich repeat receptor-like kinase (Table S8). The *XIP1* knock-down mutants shows the accumulation of cells with ectopic lignification in regions of phloem in the vascular bundles of inflorescence stems (Bryan et al., 2012).

The homeodomain containing TFs were well represented in the list of candidate genes with nine members. Members of this family have been shown to regulate procambium cell activities by promoting secondary walled xylem cell differentiation during vascular development. Some HD-ZIP III TF (HB8, PHV/HB9, PHB/HB14, REV/IFL1, and CAN/HB15) and KANADI TF (KAN1-KAN3) were shown to be involved in the secondary walled cell type formation and patterning in roots and stems (Baima et al., 2001; Emery et al., 2003; Kim et al., 2005; Ilegems et al., 2010). Three of the homeodomain TF mutants analyzed in our study exhibited SCW phenotypes. The *blh6* mutant had less lignified SCW mainly in the xylary and interfascicular fibers, whereas in the *hb5* mutant the fibers in both fascicular and interfascicular regions were heavily lignified. In the *hb15* mutant, both regions were also highly lignified but in addition ectopic lignification was observed in the parenchymatous cells adjacent to fiber and xylem cells (Figures 4B,D). This suggests that HB15 represses the SCW formation program rather than only promote the xylem cell differentiation as was concluded from earlier studies where down-regulation of *CAN/HB15* stimulated xylem production, and over-expression of a miR166-resistant *HB15* (gain-of-function mutant) resulted in reduced xylem formation (Kim et al., 2005). Co-expression analyses revealed interesting clues for *BLH6* which was co-expressed with genes involved in the biosynthesis of the three main polymers i.e., cellulose, xylan, and lignin as well as with the master regulator *MYB46* and its closest paralog *MYB83*. Together with the hypolignified phenotype of the mutant and the thinner SCW particularly in the fibers, this further supports a role of BLH6 as an activator of the whole SCW program.

*AP2 ERF* TF (AT3G14230) was identified in all four SCW-related transcriptomic datasets and exhibited high and preferential expression in xylem, but the corresponding mutant had no detectable cell wall phenotype. Twelve members of the AP2 ERF TF family were highlighted by our *in silico* approach, seven of which had high and preferential expression in xylem and another (AT5G61590) was strongly induced during *in vitro* tracheary element formation. Although this family was the second most highly represented TF family just after the MYBs in the 80 candidate list, none of its members have yet been shown to be directly involved in the regulation of SCW formation. This family therefore deserves more attention especially because it was recently reported that ethylene regulates cambium activity and promotes secondary walled xylem formation (Love et al., 2009).

Some members of the auxin-dependent TFs Aux/IAA and ARF families have been shown to be involved in vascular tissue formation. For example, loss-of-function in *ARF5/MP* (Hardtke and Berleth, 1998) and gain-of-function in *IAA12/BDL* (Hamann et al., 2002) resulted in reduced and discontinuous vascular formation. These TF families were also highly represented within the 80 candidates with seven and two members for Aux/IAA and ARF, respectively. *IAA9* was a very promising candidate found in the four transcriptomic datasets, highly and preferentially expressed in xylem and during tracheary elements differentiation. Unfortunately the corresponding mutant was unavailable at the time this work was performed. T-DNA insertion mutants corresponding to *ARF4, ARF6* and *IAA28*, and an *IAA11* RNAi transgenic line were analyzed here but did not show any obvious SCW phenotype. This is very likely due to their functional redundancy as reported in previous studies (Okushima et al., 2005; Overvoorde et al., 2005). The creation of double/triple mutants of these paralog genes might be necessary to further assess their involvement in SCW formation.

The hypolignified lines *blh6* and *zinc finger TF* displayed earlier flowering time as compared to control whereas the hyperlignification line *hb5* exhibited delayed flowering time. Two previous studies demonstrated that flowering induction time was determinant for xylem expansion and SCW formation in Arabidopsis hypocotyls and roots. Some major QTLs for SCW thickening during xylem expansion and fiber differentiation correlated tightly with a major flowering time QTL. In addition, transient induction of flowering at the rosette stage promoted SCW thickening and xylem expansion (Sibout et al., 2008). Double mutant of two flowering time genes *soc1 ful* showed a synergistically delayed flowering time and a dramatically increased SCW formation with wood development present throughout all stems and to a much larger extent than any Arabidopsis mutant described to date (Melzer et al., 2008). Collectively these results suggest that the flowering induction is coupled with the SCW thickening program and xylem formation.

In conclusion, we described here a post-genomic approach that enabled us to propose a list of 80 promising candidate genes potentially regulating SCW formation and/or lignification. Many of the available mutants analyzed did not provide any detectable SCW phenotype and complementary approaches (overexpression, using different alleles, dominant repression, or multiple mutants) are now necessary to further characterize their function. However, the six TFs of which mutants exhibited clear lignin phenotypes, further highlight the complexity of the regulatory network controlling SCW formation. Their in depth functional characterization should allow a better understanding of the regulation of lignification and SCW formation which may ultimately be used to improve the saccharification potential.

## Materials and methods

### Cross-comparison of microarray datasets

Four *in house* microarray datasets were generated in our laboratory. In brief, datasets are from *wat1* T-DNA Arabidopsis mutant CATMA microarray (Ranocha et al., 2010); EgMYB1 (Legay et al., 2010), EgMYB2 over-expressed in *Arabidopsis* (unpublished), and orthologs of *Eucalyptus* xylem expressed genes (Rengel et al., 2009). Publicly available microarray datasets were extracted from Genevestigator (https://www.genevestigator.com) (Hruz et al., 2008) by using Arabidopsis ATH1 22k array platform (7392 array datasets).

### Plant material and growth condition

The mutant lines were isolated from the T-DNA mutagenized populations in the SALK collection (Alonso et al., 2003) and from the RNAi transgenic plant populations in the Agrikola collection (http://www.agrikola.org). Seeds were obtained from the Nottingham Arabidopsis Stock Center (NASC) (http://arabidopsis.info/) and GABI (http://www.gabi-kat.de/). Homozygote lines were obtained from NASC or generated in lab and verified by PCR genotyping with gene specific primers and the respective left border primers of the T-DNA listed in supplementary Table S11. The transcript levels of each target gene in the six T-DNA insertion mutant were assessed (Figure S1) and the corresponding primers are listed in supplementary Table S12. Plants were grown in jiffy peat pellets then transferred to standard soil in culture room in short day conditions [9 h light, 200 μmol photons m^−1^s^−1^, 22°C (day)/20°C (night), 70% RH]. The flowering time was considered from sowing day until the flower stem reached 20 cm in height.

### Microscopy

The histological comparative analysis of SCW between wild type and mutants was done at the stage of newly formed green siliques, about 2 weeks after bolting, when the inflorescence stems reach 20 cm in height. At this stage, the basal part of the inflorescence stem abundantly develops cells undergoing secondary wall thickening (xylem vessel cells, fascicular, and interfascicular fiber cells). Lignin polymers are the characteristic components of SCW and are normally absent from primary cell wall, therefore we used lignin deposition detection techniques to screen for SCW phenotype. Two methods were then chosen to detect the lignin polymers in the sections for microscopic observation. Firstly we used the natural auto fluorescence of the aromatic ring moieties on the subunits of the lignin polymer under UV-light exposition. Secondly, we used the phloroglucinol-HCl coloration which stains specifically lignin polymer precursors coniferaldehyde and *p*-coumaraldehyde in the SCW giving a red-purple color when observed under normal light. Cross sections of inflorescence stems at the basal end (100–150 μm) were either observed using auto-fluorescence or stained with phloroglucinol-HCl. Auto-fluorescence was observed with a Leica microscope (excitation filter Bp 340–380 nm; suppression filter Lp 430 nm; http://leica.com). Phloroglucinol-HCl was directly applied on the slide. Images were recorded with a CCD camera (Photonic Science, http://www.photonic-science.co.uk).

### Co-expression analysis

Three co-expression analysis tools were explored using Genevestigator (https://www.genevestigator.com), Arabidopsis co-expression data mining tools (http://www.arabidopsis.leeds.ac.uk/act/), and GeneCAT (http://genecat.mpg.de/). The results were presented using Genevestigator output tables and genes classified according to gene ontology semantic (Berardini et al., 2004). We used Genevestigator Arabidopsis ATH1 22k array platform with *in absentia* parameters that comprise all 7392 qualified datasets and is regardless of the underlying microarray datasets and the bait genes (i.e., all samples, condition-independent, and no-tissues specific bait genes), 50 was as “cut-off” threshold for co-expressed genes list.

### Conflict of interest statement

The authors declare that the research was conducted in the absence of any commercial or financial relationships that could be construed as a potential conflict of interest.
