# Erratum: Identification of novel transcription factors regulating secondary cell wall formation in *Arabidopsis*

**DOI:** 10.3389/fpls.2014.00246

**Published:** 2014-06-03

**Authors:** Hua Cassan-Wang, Nadia Goué, Mohammed N. Saidi, Sylvain Legay, Pierre Sivadon, Deborah Goffner, Jacqueline Grima-Pettenati

**Affiliations:** Laboratoire de Recherche en Sciences Végétales, UMR5546, Centre National de la Recherche Scientifique, Université Toulouse III (UPS)Toulouse, France

**Keywords:** secondarycellwall, xylem, fibers, transcription factors, arabidopsis, lignin, co-expression, biofuels

After our article was published on line, we noticed that a typographical mistake occured in the name of an Arabidopsis mutant:

p 6, in the results, last paragraph of the section ≪ phenotypes of the TF T-DNA mutant or RNAi transgenic lines ≫, One should read *hb5* instead of *hb15*

Here is the corrected text

The overall growth behavior of the mutants did not differ significantly from the controls, except the bolting and flowering time were altered in three of the mutant lines. Hypolignified *blh6* and *zinc finger* lines bolted and flowered earlier than controls (Figures 5A,C) whereas the hyperlignified ***hb5*** line exhibited delayed bolting and flowering (Figures 5B,C). In addition, ***hb5*** mutants exhibited aerial rosettes at the base of the lateral inflorescence branches instead of growing cauline leaves as in wild-type plants (Figure 5D).

## Conflict of interest statement

The authors declare that the research was conducted in the absence of any commercial or financial relationships that could be construed as a potential conflict of interest.

