# Supplementary material for: Identification of novel transcription factors regulating secondary cell wall formation in Arabidopsis
Source: Front Plant Sci. 2013 Jun 11;4:189. doi: 10.3389/fpls.2013.00189 (PMC3677987; doi:10.3389/fpls.2013.00189)
Supplement: Supplementary file 12 [file DataSheet12.PDF]

A

B

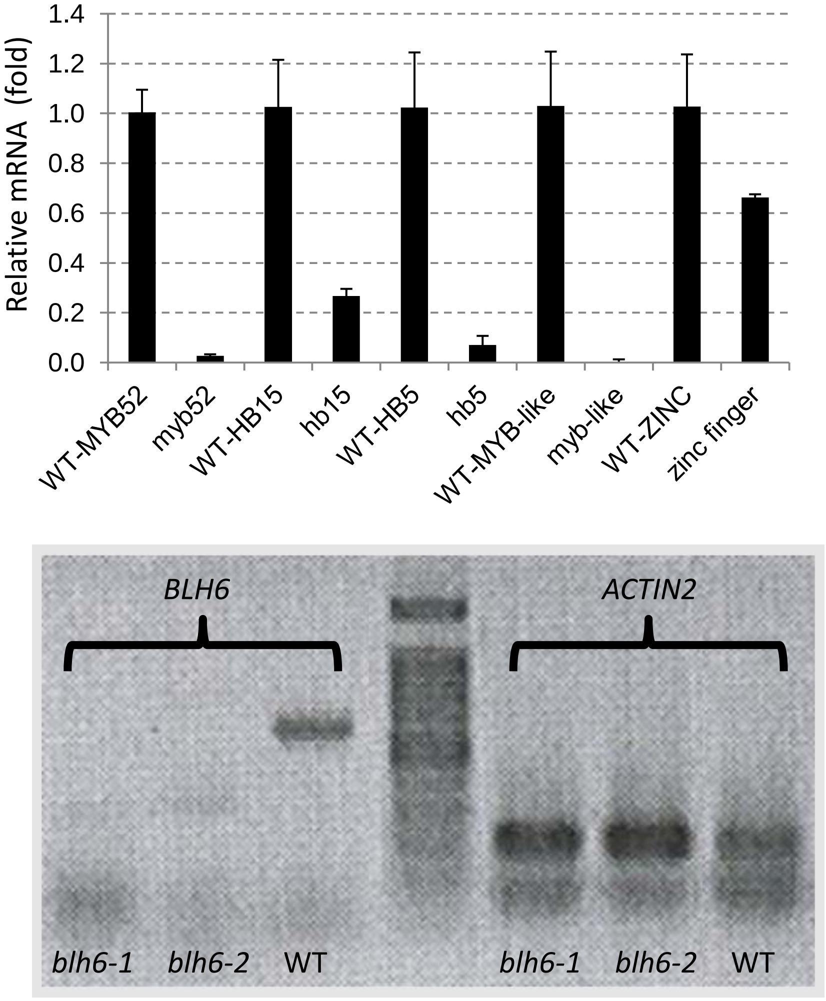

Figure S1. Transcript levels of T-DNA mutated genes.
A) RT-qPCR analysis of the transcript levels of *MYB52*, *HB15*, *HB5*, *MYB-Like* and *ZINC FINGER* in wild-type plants and T-DNA mutants. The relative expression levels of the corresponding genes were determined through the $2^{-\Delta\Delta CT}$ method using *ubiquitin10* as a reference gene. The expression levels in wild-type were set to 1.0. **B)** RT-PCR analysis of the *BLH6* transcript level in *blh6* T-DNA lines and in wild-type plants. *ACTIN2* was used to internal reference gene. The primers of corresponding genes are presented in Table S12.
